# Closing the diagnostic gap in transthyretin cardiac amyloidosis: red flags, modern imaging, and emerging therapies

**DOI:** 10.3389/fcvm.2026.1935800

**Published:** 2026-09-29

**Authors:** Zhengjun Yi, Peng Li, Xueqian Wang

**Affiliations:** 1 Department of Cardiovascular Medicine, Fuling People’s Hospital of Chongqing, Chongqing, China; 2 Department of Cardiovascular Medicine, Meishan People’s Hospital, Meishan, China

**Keywords:** acoramidis, bone scintigraphy, diagnostic algorithm, diagnostic delay, HFPEF, tafamidis, transthyretin cardiac amyloidosis, vutrisiran

## Abstract

Transthyretin cardiac amyloidosis (ATTR-CM) is an increasingly recognized, treatable, and still underdiagnosed cause of heart failure with preserved ejection fraction (HFpEF) and unexplained left ventricular hypertrophy in older adults, with a typical diagnostic delay of several years. We conducted a narrative review with a systematic literature search of PubMed and Embase (January 2000 to June 2026) using database-specific controlled vocabulary and free-text terms related to ATTR cardiomyopathy, clinical red flags, bone scintigraphy, monoclonal protein testing, cardiac magnetic resonance, tafamidis, acoramidis, vutrisiran, and emerging TTR-directed therapies. Two authors screened titles and abstracts, resolved disagreements by consensus, and prioritized multicenter cohorts, clinical trials, meta-analyses, systematic reviews, and guideline or consensus documents. Sixty-two references were included. Three practice points emerge from the literature. First, suspicion should be triggered by red flags such as bilateral carpal tunnel syndrome, lumbar spinal stenosis, spontaneous distal biceps tendon rupture, and the combination of increased ventricular wall thickness with low or normal QRS voltage. Second, the contemporary diagnostic pathway requires parallel bone scintigraphy and complete monoclonal protein screening; grade 2-3 myocardial uptake supports non-biopsy diagnosis of ATTR-CM only when monoclonal screening is negative and myocardial uptake is confirmed, preferably by SPECT or SPECT/CT. Third, treatment options now extend beyond tafamidis to acoramidis and vutrisiran, with amyloid-depleting antibodies under active investigation. We propose a pragmatic, resource-stratified diagnostic framework for targeted case-finding rather than population-level screening.

## Introduction

Transthyretin cardiac amyloidosis (ATTR-CM) is a common, treatable, and substantially underdiagnosed cause of heart failure with preserved ejection fraction (HFpEF) in older adults. The mean diagnostic delay of 2–4 years reflects a recurring clinical pattern: patients consult multiple specialists—orthopedics for carpal tunnel syndrome, neurosurgery for spinal stenosis, cardiology for dyspnea—before the unifying diagnosis is made. This review synthesizes current evidence on clinical red flags, modern non-invasive diagnostic pathways, and emerging therapies for general cardiologists.

Cardiac amyloidosis results from misfolded protein fibrils deposited in the myocardium, leading to thickening of the walls, diastolic dysfunction, and eventually heart failure (1). Among more than 30 amyloidogenic proteins, two account for almost all clinically significant cardiac involvement: immunoglobulin light-chain (AL) amyloidosis and transthyretin (ATTR) amyloidosis (2). ATTR in particular has shifted from a rare, untreatable condition to one that is commonly missed but increasingly manageable (3).

Two forms exist. Wild-type ATTR (ATTRwt), once called senile systemic amyloidosis, arises from age-related misfolding of normal TTR protein and mostly affects men over 70 (4). Variant ATTR (ATTRv) results from autosomal dominant TTR mutations, with founder mutations such as V122I (common in people of African descent), T60A (United Kingdom and Ireland), and V30M (Portugal, Sweden, Japan) (5). ATTRv-CM can manifest earlier, sometimes in the fifth decade.

Recent estimates indicate ATTR-CM in 10%–15% of HFpEF patients over 60 (6, 7), 13%–16% of severe aortic stenosis patients undergoing transcatheter aortic valve replacement (8, 9), and approximately 5% of hospitalized heart failure patients (10). Yet fewer than 2% of estimated cases in the United States have received a formal diagnosis (11). Patients experience a 2–4 year diagnostic delay, consulting multiple physicians before the correct diagnosis is established (12), during which amyloid burden accumulates and diminishes therapeutic efficacy (13).

In this review, we cover the clinical clues that should trigger suspicion, the modern non-invasive diagnostic workup, common diagnostic pitfalls, and the expanding therapeutic landscape. Because several recent guidelines and consensus statements already provide comprehensive recommendations, the specific contribution of this article is practical rather than directive: we synthesize red flags, monoclonal protein testing, bone scintigraphy with SPECT/SPECT-CT confirmation, cardiac magnetic resonance, and treatment selection into a resource-stratified framework for general cardiologists. The aim is to support targeted case-finding in patients with compatible clinical phenotypes, not to advocate indiscriminate population screening.

## Methods

We searched PubMed and Embase for articles published between January 2000 and June 2026. PubMed searches combined MeSH terms and free-text terms including “transthyretin amyloid cardiomyopathy,” “ATTR cardiomyopathy,” “cardiac amyloidosis,” “heart failure with preserved ejection fraction,” “hypertrophic cardiomyopathy,” “carpal tunnel syndrome,” “biceps tendon rupture,” “bone scintigraphy,” “technetium pyrophosphate,” “cardiac magnetic resonance,” “tafamidis,” “acoramidis,” “vutrisiran,” “patisiran,” “eplontersen,” “coramitug,” and “cliramitug.” Embase searches used corresponding Emtree terms and free-text terms. The final search was performed in June 2026. We included original research, phase II-III trials, meta-analyses, systematic reviews, and guideline or expert consensus documents from major cardiovascular, nuclear cardiology, and amyloidosis societies. Case reports, non-English publications, and conference abstracts without full text were excluded. Small case series were generally not prioritized for prevalence or treatment-effect estimates, but clinically important small studies were considered when they addressed rare variants, imaging pitfalls, or extracardiac red flags not adequately captured in larger cohorts. Reference lists of included articles were hand-searched. The final reference list comprises 62 citations selected for relevance to practical diagnosis, diagnostic pitfalls, and disease-modifying therapy.

This is a narrative review with a systematic literature search rather than a systematic review or meta-analysis. Because the literature combines heterogeneous screening cohorts, diagnostic endpoints, imaging protocols, and treatment populations, we did not pool estimates statistically. We instead prioritized large multicenter cohorts, prospective trials, systematic reviews, and contemporary guideline or consensus documents, while using smaller studies selectively for rare but clinically important diagnostic issues. The search strategy was designed to be transparent, but the final synthesis remains narrative in scope.

## Epidemiology and the diagnostic gap

### Prevalence

Prevalence estimates should be interpreted according to the population screened and the diagnostic strategy used. In selected HFpEF cohorts, bone scintigraphy-based studies have reported ATTR-CM in a meaningful minority of older patients, but estimates vary with age, wall-thickness criteria, inpatient versus outpatient recruitment, and whether SPECT or SPECT/CT confirmation is required (6, 7, 10, 14). In the multicenter PRACTICA study, consecutive patients aged >=50 years with HFpEF and left ventricular hypertrophy were evaluated across 20 Spanish hospitals, illustrating the importance of interpreting prevalence within a defined high-risk phenotype (15).

ATTR-CM also emerges in other clinical contexts, but these settings are not interchangeable. In severe aortic stenosis cohorts referred for transcatheter aortic valve replacement, ATTR-CM is found in approximately 13%–16% (8, 9). Among older patients with an unexplained hypertrophic cardiomyopathy phenotype, the multicenter TTRACK study identified ATTR-CM in approximately 19%, supporting ATTR-CM as an important differential diagnosis in late-onset unexplained left ventricular hypertrophy (16). By contrast, TTR amyloid detected in tenosynovial tissue at carpal tunnel release reflects extracardiac amyloid deposition and does not by itself establish clinically overt cardiac amyloidosis (17, 18, 61).

### Diagnostic delay

A survey of 587 ATTR-CM patients reported a mean delay from first symptom to diagnosis of 3.6 years for ATTRwt and 4.2 years for ATTRv, with patients consulting a median of five physicians (12). The THAOS registry corroborates these findings (19). Untreated, median survival is approximately 3.6 years for ATTRwt and 2.5 years for ATTRv-CM (6). NYHA class and NT-proBNP at diagnosis are strong independent predictors of outcome, and earlier diagnosis improves prognosis substantially (20, 21).

### Strategy for earlier detection

A practical approach to reducing diagnostic delay is best framed as targeted case-finding. In selected high-risk groups, such as older patients with HFpEF and increased wall thickness, low-flow low-gradient aortic stenosis, unexplained late-onset hypertrophic phenotypes, or bilateral carpal tunnel syndrome plus cardiac symptoms, structured evaluation can increase diagnostic yield. This should be distinguished from formal population-level screening programs, for which evidence on implementation, cost-effectiveness, and outcome benefit remains incomplete.

### Extracardiac red flags

ATTR-CM is a systemic disease. Before the heart becomes thickened and stiff, amyloid deposits in tendons, ligaments, and soft tissues, providing the best opportunity for early diagnosis.

### Carpal tunnel syndrome

Bilateral carpal tunnel syndrome (CTS) is the earliest and most reliable clue. TTR amyloid deposits in the flexor retinaculum and tenosynovium, causing bilateral CTS that precedes cardiac diagnosis by 5–8 years (22, 23). Among ATTRwt-CM patients, over half report bilateral CTS and 30%–40% have undergone carpal tunnel surgery (12, 24). The CTS is typically bilateral (not limited to the dominant hand), frequently requires surgical release, and can recur postoperatively. A retrospective study of 607 patients undergoing carpal tunnel release identified TTR amyloid in up to 34% of those over 70 years and over 50% when both hands were affected (18).

Clinically, any man over 65 with bilateral CTS and suggestive cardiac symptoms should undergo screening. A history of bilateral carpal tunnel release surgery is an even stronger indication for screening.

### Lumbar spinal stenosis

TTR deposits in the ligamentum flavum cause lumbar spinal stenosis in 15%–30% of ATTR-CM patients, typically presenting several years before cardiac diagnosis (25, 26). A patient who has undergone both carpal tunnel release and lumbar spine surgery before developing heart failure represents a classic presentation that should prompt diagnostic testing.

### Biceps tendon rupture

Spontaneous rupture of the distal biceps tendon has been reported as an additional red flag. It is less sensitive than carpal tunnel syndrome but may be relatively specific when present, particularly in men with an otherwise unexplained hypertrophic phenotype or other tenosynovial manifestations (27–29).

### Aortic stenosis and amyloidosis

ATTR-CM is identified in 13%–16% of patients undergoing transcatheter aortic valve replacement (8, 9). Conversely, approximately 10%–15% of ATTR-CM patients have moderate-to-severe aortic stenosis (30). This relationship is bidirectional: ATTR-CM stiffens the ventricle and reduces stroke volume, lowering the transvalvular gradient even in the presence of truly severe valve stenosis (31). This creates “low-flow, low-gradient” physiology with preserved ejection fraction, complicating echocardiographic assessment of aortic stenosis severity. Any elderly patient with low-gradient severe aortic stenosis and preserved left ventricular function should undergo ATTR-CM screening.

### Neuropathy and autonomic dysfunction

Peripheral neuropathy and autonomic dysfunction are more common in ATTRv than ATTRwt, but both forms can involve the nervous system (32). Orthostatic hypotension (systolic pressure drop ≥20 mm Hg from supine to standing), erectile dysfunction, gastroparesis, and neurogenic bladder are potential presentations (33). Orthostatic vital signs and targeted clinical questions can identify these findings during routine office evaluation.

### Pattern recognition

The most suggestive clinical constellation—in a man over 65 with HFpEF—comprises three findings: bilateral CTS (particularly if surgically treated), lumbar spinal stenosis, and left ventricular hypertrophy with low or normal QRS voltage. While not every patient exhibits all three findings, their combination should lower the threshold for diagnostic testing.

### Diagnostic workup

The diagnostic workup for suspected ATTR-CM follows a stepwise approach integrating electrocardiography (ECG), echocardiography with global longitudinal strain (GLS), bone scintigraphy, monoclonal protein screening, cardiac magnetic resonance (CMR), and genetic testing.

### Echocardiography and electrocardiography

Echocardiography is typically the initial diagnostic test. Increased left ventricular wall thickness (>=12 mm), biatrial enlargement, valve thickening, restrictive filling physiology, and pericardial effusion can support suspicion. A granular or “speckled” myocardial appearance has historically been described, but it is neither sensitive nor specific in the era of harmonic imaging and should not be used as a decisive diagnostic criterion. Relative apical sparing on global longitudinal strain (GLS), often expressed as an apical-to-basal strain ratio ≥1.0, is a useful supportive sign, but it is not pathognomonic and may be seen in both ATTR and AL cardiac amyloidosis (34, 35, 62).

The electrocardiogram reveals a discordant finding: low or normal QRS voltage despite echocardiographically thickened ventricles. This “low-voltage hypertrophy” mismatch is present in approximately 20%–30% of ATTRwt cases, 40%–50% of ATTRv cases, and 50%–60% of AL amyloidosis cases (36, 37). Its absence does not exclude the diagnosis, particularly in ATTRwt.

### Bone scintigraphy

Bone scintigraphy with technetium-labeled bisphosphonates (99mTc-PYP in the United States; 99mTc-DPD or 99mTc-HMDP in Europe and Asia) has transformed ATTR-CM diagnosis. The Perugini grading system scores myocardial uptake from 0 (none) to 3 (intense uptake greater than bone). Planar imaging alone can be misleading because blood-pool activity may mimic myocardial uptake. SPECT or SPECT/CT should therefore be performed whenever possible to confirm true myocardial tracer uptake and to reduce false-positive interpretation (38–41).

When Perugini grade 2 or 3 myocardial uptake is present and monoclonal protein screening is negative, specificity for ATTR-CM exceeds 99%, and biopsy is not required (Class I, Level B) (39, 40).

Important false-positive and false-negative scenarios are summarized in Table 1. False positives include persistent blood-pool activity, AL amyloidosis, recent myocardial infarction, rib fracture overlying the heart, hydroxychloroquine cardiotoxicity, and rare non-TTR amyloid types. False negatives may occur with early or low-burden disease, suboptimal acquisition, and selected ATTRv variants whose fibrils show little or no tracer avidity.

**Table 1:** Causes of false-positive and false-negative bone scintigraphy in suspected ATTR-CM.

Scenario	Examples	Practical implication
False-positive or overcalled uptake	Blood-pool activity on planar imaging	Confirm myocardial uptake with SPECT or SPECT/CT.
False-positive or non-ATTR uptake	AL amyloidosis; rare non-TTR amyloid types	Complete monoclonal protein screening is mandatory; biopsy and amyloid typing are required if monoclonal testing is abnormal.
Regional uptake unrelated to ATTR-CM	Recent or subacute myocardial infarction; rib fracture overlying the cardiac silhouette	Interpret uptake pattern with clinical history and cross-sectional localization.
Drug-related mimic	Hydroxychloroquine cardiotoxicity	Consider medication history and alternative cardiomyopathy evaluation.
False-negative scan	Early or low-burden ATTR-CM	Proceed to CMR or biopsy if suspicion remains high.
Variant-associated false negative	Selected ATTRv variants including Phe64Leu and Ser77Tyr, with occasional reports in Val30Met	A negative scan does not exclude ATTRv-CM in a compatible phenotype or genotype.
Technical false negative	Suboptimal acquisition timing or protocol variation	Use standardized acquisition and expert nuclear cardiology interpretation.

### Monoclonal protein screening

Complete monoclonal screening comprises three tests: serum protein electrophoresis with immunofixation, serum free light chain assay with kappa/lambda ratio, and 24-hour urine immunofixation (42).

An abnormal free light chain ratio (interpreted with attention to renal function) and/or positive serum or urine immunofixation raises the possibility of AL amyloidosis and fundamentally changes the diagnostic pathway. A positive monoclonal screen does not automatically establish AL amyloidosis, because monoclonal gammopathy of undetermined significance is common in older patients and may coexist with ATTR-CM. In this scenario, hematology consultation and tissue confirmation with amyloid typing, preferably by mass spectrometry when available, are required to avoid misclassification and delayed treatment of AL amyloidosis (43). Cardiac magnetic resonance cannot reliably distinguish AL from ATTR, and monoclonal-positive patients should not be routed through advanced imaging as a substitute for tissue typing.

If monoclonal screening is negative and bone scintigraphy demonstrates grade 2–3 uptake, ATTR-CM is confirmed and disease-modifying therapy can be initiated.

### Cardiac magnetic resonance

CMR is most useful when monoclonal screening is negative and bone scintigraphy is grade 0-1, or when echocardiographic findings remain unexplained. Typical findings include difficulty nulling the myocardium on inversion-time scout imaging, diffuse subendocardial or transmural late gadolinium enhancement, elevated native T1, and expanded extracellular volume fraction (44–46). Native T1 and extracellular volume fraction can help distinguish cardiac amyloidosis from several other causes of increased wall thickness, but they cannot reliably determine amyloid subtype. For patients with renal impairment who cannot receive gadolinium-based contrast, native T1 mapping alone provides useful diagnostic information. CMR also offers prognostic value: late gadolinium enhancement extent and extracellular volume fraction correlate with clinical outcomes (46).

### Genetic testing and biopsy

All patients diagnosed with ATTR-CM should undergo TTR gene sequencing to distinguish ATTRwt from ATTRv (Class I, Level C) (40). However, treatment should not be delayed pending genetic results: disease-modifying therapy should be initiated upon diagnosis. The genetic result guides prognosis, informs therapeutic selection (gene-silencing therapy may be preferred for ATTRv), and enables family screening. For ATTRv patients, genetic counseling and cascade testing of first-degree relatives is recommended, with age of initiation tailored to the specific mutation and family phenotype (generally 10 years before the earliest-onset family case, or between 20 and 25 years for high-penetrance mutations such as V30M) (Class I, Level C) (47). For ATTRwt patients, cascade family screening is not recommended, as ATTRwt is a non-hereditary condition.

When bone scintigraphy and CMR are inconclusive, or when monoclonal screening is positive (with direct referral to biopsy as described above), endomyocardial biopsy with mass spectrometry-based typing remains the diagnostic gold standard (48). Abdominal fat pad aspiration can be attempted first given its lower invasiveness, but its sensitivity is only 50%–70% (49).

### Prognostic assessment

NYHA class, NT-proBNP, renal function, and troponin are central to prognostic assessment. Two staging systems are commonly used (Table 2). The National Amyloidosis Centre (NAC) ATTR staging system uses NT-proBNP and estimated glomerular filtration rate, whereas the Mayo ATTR system uses NT-proBNP and cardiac troponin T or high-sensitivity troponin T (20, 50). These systems support risk stratification and follow-up but should be interpreted in clinical context, because atrial fibrillation, kidney disease, and age can influence biomarker levels.

**Table 2:** Common staging systems for ATTR-CM.

System	Biomarkers	Stage definition	Clinical use
NAC ATTR staging	NT-proBNP and eGFR	Stage I: neither NT-proBNP >3,000 ng/L nor eGFR <45 mL/min/1.73 m2; Stage II: one criterion; Stage III: both criteria	Simple risk stratification using cardiac stress and renal function.
Mayo ATTR staging	NT-proBNP and cardiac troponin T or high-sensitivity troponin T	Stage I: neither biomarker above threshold; Stage II: one biomarker above threshold; Stage III: both biomarkers above threshold	Risk stratification using cardiac stress and myocardial injury.
Interpretation caveat	All biomarker-based systems	Biomarkers may be influenced by atrial fibrillation, renal dysfunction, age, and intercurrent illness	Use alongside NYHA class, imaging burden, genotype, comorbidities, and treatment goals.

## Therapeutic options

### TTR stabilizers

Tafamidis is first-line therapy for ATTR-CM (Class I, Level B). The ATTR-ACT trial (*n* = 441) demonstrated lower all-cause mortality [hazard ratio [HR] 0.70, 95% confidence interval [CI] 0.51–0.96] and fewer cardiovascular hospitalizations at 30 months (20). Long-term follow-up confirmed sustained benefit (21), and treatment initiation within 6 months of diagnosis was associated with improved survival (adjusted HR 0.57) (51). Periodic liver enzyme monitoring is recommended, as asymptomatic transaminase elevation occurs in approximately 2%–3% of patients.

Acoramidis improved clinical outcomes in the ATTRibute-CM trial and may be considered as an alternative TTR stabilizer to tafamidis in appropriate patients (52). Formal incorporation into major guideline recommendation tables remains evolving, and no head-to-head comparison with tafamidis is available.

### Gene silencing therapies

Vutrisiran has demonstrated the most robust efficacy among RNA-interfering agents. In the HELIOS-B trial (*n* = 655), vutrisiran reduced the composite of all-cause mortality and cardiovascular hospitalization by 28% (HR 0.72, 95% CI 0.56–0.93) and received FDA approval for ATTR-CM in April 2026 (Class IIa, Level B) (53). It is administered as a subcutaneous injection every three months. Injection site reactions occur in approximately 4%–6% of patients but are generally mild.

For patients with ATTRv-CM, particularly those with concomitant polyneuropathy, vutrisiran should be strongly considered as frontline therapy given its dual cardiac and neurological benefit. The HELIOS-B trial demonstrated improvements in both the composite cardiovascular endpoint and the modified Neuropathy Impairment Score (53).

Patisiran, the predecessor RNAi agent, preserved functional capacity in the APOLLO-B trial (*n* = 360) but did not meet the primary composite endpoint (54); it requires intravenous administration and premedication for infusion reactions and is now largely superseded by vutrisiran in the cardiac context.

Eplontersen, a GalNAc-conjugated antisense oligonucleotide, is under investigation for ATTR-CM in the CARDIO-TTRansform program. Evidence from NEURO-TTRansform supports its activity in hereditary transthyretin amyloidosis with polyneuropathy, but those data should not be cited as completed ATTR-CM outcome evidence (55).

### Comprehensive management of heart failure symptoms

In addition to disease-modifying therapies, cautious symptom management is essential as ATTR-CM patients have unique hemodynamic vulnerabilities.

**Diuretic therapy.** ATTR-CM ventricles are stiff and preload-dependent. Loop diuretics should be initiated at low doses and titrated gradually to avoid precipitous preload reduction leading to prerenal azotemia and hypotension. Patients frequently require lower doses than those tolerated in other HFpEF phenotypes.

**Neurohormonal antagonists.** Angiotensin-converting enzyme inhibitors, angiotensin receptor blockers, and beta-blockers are often poorly tolerated due to baseline hypotension (common in ATTR-CM from autonomic dysfunction and reduced stroke volume). These agents should be initiated at low doses only when strictly indicated (e.g., concomitant hypertension or coronary artery disease) and up-titrated cautiously with close blood pressure monitoring.

**Atrial fibrillation management.** Atrial fibrillation occurs in 40%–60% of ATTR-CM patients. Direct oral anticoagulants (DOACs) are preferred over warfarin given the lower bleeding risk profile, including a lower incidence of intracranial hemorrhage. Despite theoretical concerns regarding gastrointestinal bleeding from amyloid infiltration, real-world data support DOAC safety in this population. Rate control is preferred over rhythm control; beta-blockers and calcium channel blockers should be used cautiously given the risk of hypotension. Amiodarone is the safest antiarrhythmic for rhythm control if required.

**Device therapy.** Pacemaker implantation is indicated for symptomatic bradycardia or high-degree atrioventricular block, which are common due to amyloid infiltration of the conduction system. However, implantable cardioverter-defibrillators (ICDs) for primary prevention are of uncertain benefit (Class IIb, Level C per 2023 ESC guidelines); sudden death in ATTR-CM is predominantly due to pulseless electrical activity rather than ventricular tachyarrhythmias. The decision for ICD implantation should be individualized.

### Gene-tailored selection of disease-modifying therapy

The choice among available disease-modifying therapies should incorporate genotype, neuropathy status, and disease stage.

For patients with ATTRwt (wild-type) and early-stage ATTRv without significant neuropathy, tafamidis remains the first-line stabilizer given its robust long-term survival data from the ATTR-ACT trial and extended follow-up (20, 21). Acoramidis is a reasonable alternative, showing more rapid NT-proBNP reduction in the ATTRibute-CM trial, though no head-to-head comparison with tafamidis exists (52).

For patients with ATTRv-CM, particularly those with concomitant polyneuropathy, vutrisiran should be strongly considered as frontline therapy, as it addresses both cardiac and neurological endpoints (53). Tafamidis may be added to vutrisiran for additive cardiac stabilization in advanced-stage patients, although evidence for combination therapy remains limited.

All patients initiated on disease-modifying therapy require baseline and serial assessments of NT-proBNP, estimated glomerular filtration rate, NYHA functional class, and echocardiographic parameters every 6–12 months to monitor therapeutic response.

### Emerging approaches

CRISPR-Cas9 gene editing (NTLA-2001) has demonstrated proof of concept in ATTR amyloidosis and remains under investigation for broader cardiac indications (56). Amyloid-depleting monoclonal antibodies are another emerging strategy. Coramitug showed signals of biological activity in a phase 2 randomized trial in ATTR-CM, and cliramitug showed a favorable safety profile with dose- and time-dependent reductions in surrogate markers of cardiac amyloid burden in early-phase studies; both approaches require confirmation in larger phase 3 outcome trials (57, 58). Liver transplantation, historically the only available treatment for ATTRv, has been largely supplanted by pharmacological therapy but retains a role in carefully selected early-stage patients without significant cardiac involvement (59). Diflunisal is an off-label TTR stabilizer with randomized evidence in ATTRv polyneuropathy, but it is not guideline-preferred for ATTR-CM. It may remain a selected option where approved ATTR-CM therapies are unavailable or unaffordable, with careful monitoring for renal, gastrointestinal, bleeding, and fluid-retention risks (60).

### Diagnostic algorithm for clinical practice

From the evidence reviewed, we propose the diagnostic algorithm illustrated in Figure 1. The algorithm is intended for targeted case-finding in patients with compatible clinical phenotypes, including men aged 65 years or older with HFpEF and increased wall thickness, bilateral CTS especially if surgically treated, unexplained late-onset left ventricular hypertrophy or hypertrophic cardiomyopathy phenotype, and low-flow low-gradient aortic stenosis.

**Figure 1:**
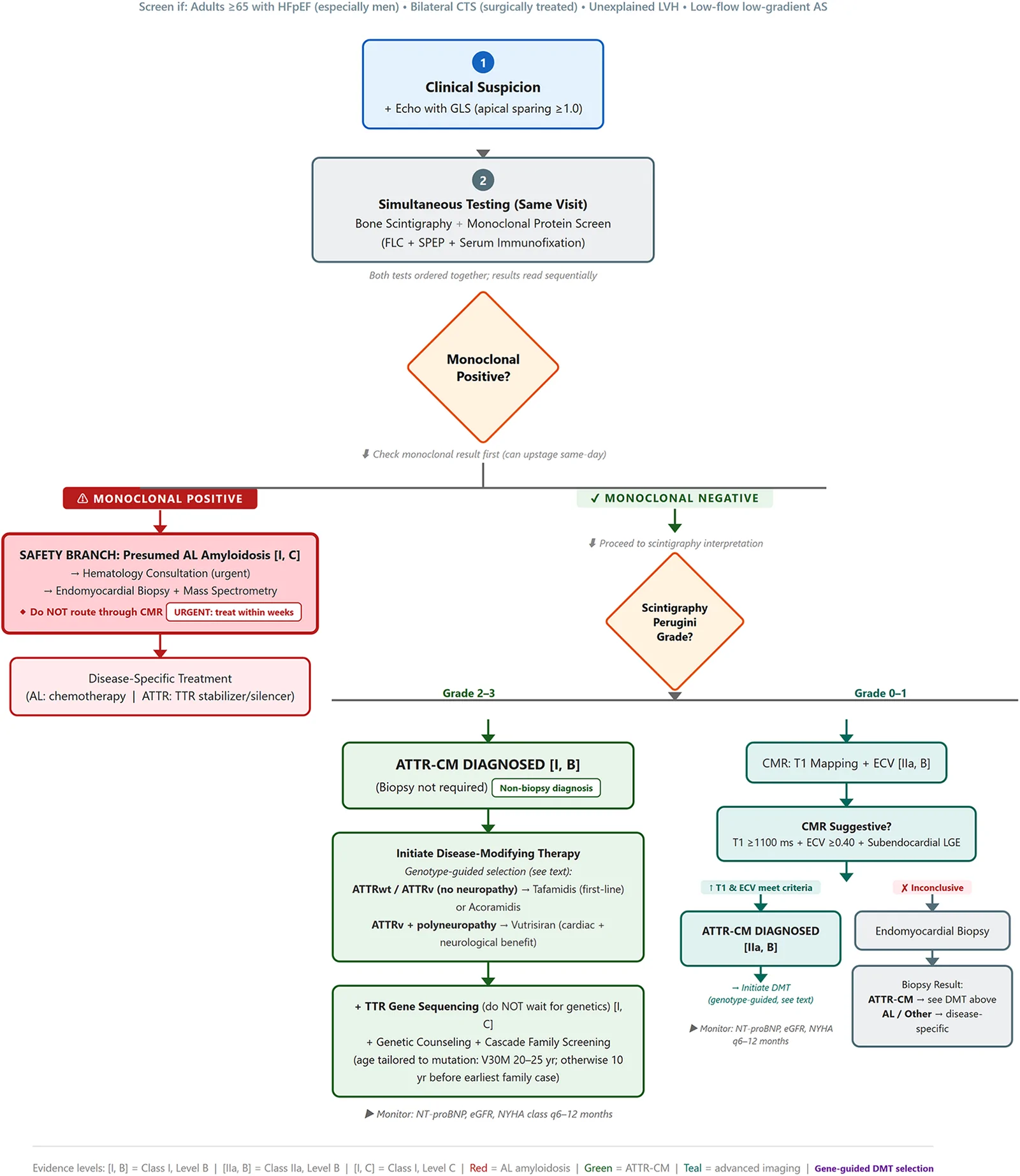
Diagnostic algorithm for ATTR cardiac amyloidosis. Sequential decision-making pathway integrating clinical red flags, bone scintigraphy, monoclonal protein screening, cardiac magnetic resonance, and genetic testing. This tiered algorithm is designed for different healthcare settings: primary hospitals without nuclear imaging can initiate the simplified three-step screening workup (ECG, echocardiography with GLS, serum free light chain assay) before referral to tertiary centers for confirmatory bone scintigraphy. Algorithm annotated with guideline evidence levels (Class of Recommendation, Level of Evidence).

The algorithm follows a sequential logic:

1. Clinical history and red flag assessment
2. Echocardiography with GLS
3. Simultaneous monoclonal protein screening and bone scintigraphy
4. Result-driven management decisions

If bone scintigraphy demonstrates grade 2–3 uptake and monoclonal screening is negative, ATTR-CM is confirmed. Disease-modifying therapy should be initiated without waiting for genetic results. TTR gene sequencing should be obtained concurrently, with subsequent genetic counseling and cascade family screening (Class I, Level B).

If monoclonal screening is positive at any scintigraphy grade, the result should not be assumed to represent AL amyloidosis, nor should ATTR-CM be excluded. Because MGUS and ATTR-CM can coexist in older patients, hematology review and tissue biopsy with amyloid typing are required before assigning amyloid subtype.

If bone scintigraphy is grade 0–1 and monoclonal screening is negative, proceed to CMR with T1 mapping and extracellular volume fraction assessment (Class IIa, Level B). If CMR is inconclusive, endomyocardial biopsy should be performed.

For settings without access to nuclear scintigraphy or CMR, a simplified referral approach using widely available tests is feasible: electrocardiography for low-voltage hypertrophy mismatch, echocardiography with GLS for relative apical sparing, and complete monoclonal protein screening. Positive clinical or imaging suspicion should prompt referral to a center with bone scintigraphy including SPECT/SPECT-CT and access to tissue typing when needed.

### Differential diagnosis

The differential diagnosis of increased left ventricular wall thickness in older adults includes hypertensive heart disease, hypertrophic cardiomyopathy, aortic stenosis-related remodeling, cardiac amyloidosis (both types), and—less commonly—Fabry disease or cardiac sarcoidosis. Table 3 summarizes the distinguishing features. The most urgent distinction is between ATTR-CM and AL amyloidosis, given the radically different prognosis and treatment approach.

**Table 3:** Differential diagnosis of increased left ventricular wall thickness in older adults.

Parameter	ATTR-CM	AL	HTN LVH	HCM	Fabry	Sarcoidosis
ECG voltage	Low-normal	Low (50%–60%)	High	High	Variable; conduction abnormalities and ventricular arrhythmias may occur	Variable (conduction defects)
LV wall thickness	Symmetric	Symmetric	Symmetric	Asymmetric (septal)	Symmetric	Focal/anomalous
GLS	Relative apical sparing common; supportive, not specific	Relative apical sparing may occur; strain does not reliably distinguish AL from ATTR	Basal-mid down, ratio <1.0	Normal/reduced basal	Basal-mid down (inferolateral)	Focal down
Native T1 (CMR)	Markedly ↑↑	Markedly ↑↑	Normal–mild ↑	Normal	Low T1	Normal–mild ↑
ECV	≥0.40	≥0.40	<0.30	<0.30	<0.30	Normal–mild ↑ (focal or diffuse, depending on involvement)
Bone scan	Grade 2–3	Grade 0–1 (rarely 2)	Grade 0	Grade 0	Grade 0	Grade 0
Bilateral CTS	Very common	Rare	Absent	Absent	Common (early)	Absent
Monoclonal protein	Negative	Positive (>95%)	Negative	Negative	Negative	Negative
NT-proBNP/wall thickness	Low–moderate	High (often >100)	Low	Low	Low	Variable
Progression	Slow (years)	Rapid (months)	Very slow	Slow	Slow	Variable

ATTR-CM, transthyretin cardiac amyloidosis; AL, immunoglobulin light-chain amyloidosis; HTN, hypertensive; LVH, left ventricular hypertrophy; HCM, hypertrophic cardiomyopathy; ECG, electrocardiogram; LV, left ventricular; GLS, global longitudinal strain; CMR, cardiac magnetic resonance; ECV, extracellular volume fraction; CTS, carpal tunnel syndrome; NT-proBNP, N-terminal pro-B-type natriuretic peptide.

Several features can raise suspicion for one subtype over another, but no clinical, echocardiographic, or CMR finding reliably distinguishes AL from ATTR cardiac amyloidosis. AL amyloidosis often progresses more rapidly and may demonstrate higher NT-proBNP relative to wall thickness; ATTRwt more often presents in older men with slower progression and tenosynovial manifestations. Low QRS voltage is less common in ATTRwt than in AL (36, 37). Bone scintigraphy grade 2-3 uptake strongly supports ATTR-CM only when complete monoclonal screening is negative and myocardial uptake is confirmed; if a monoclonal abnormality is present, tissue confirmation with amyloid typing is required (38–40).

### Diagnostic pitfalls

Clinical experience has identified several diagnostic traps. First, a normal free light chain ratio does not completely exclude AL amyloidosis: approximately 5% of AL patients have a normal kappa/lambda ratio with positive immunofixation alone, underscoring the importance of the complete panel (serum protein electrophoresis with immunofixation, free light chain, and urine immunofixation).

Second, ATTRwt can present without classic red flags. Approximately 20% of patients have no history of CTS or spinal stenosis. In these patients, the disease is discovered incidentally on echocardiography performed for other indications, and the absence of red flags should not delay diagnostic workup.

Third, Perugini grade 2 uptake can occasionally occur in AL amyloidosis or be mimicked by blood-pool activity on planar imaging. SPECT or SPECT/CT confirmation and a complete monoclonal screen are essential counterchecks; if diagnostic uncertainty persists, tissue biopsy remains definitive.

Fourth, fat pad aspiration is a reasonable initial biopsy approach given its lower invasiveness, but a negative result does not exclude cardiac involvement. If clinical suspicion remains high, proceed to endomyocardial biopsy.

## Conclusion

ATTR cardiac amyloidosis is an increasingly recognized, treatable, and still underdiagnosed condition. The persistent diagnostic delay reflects low awareness of extracardiac red flags, limited access to confirmatory imaging and amyloid typing, and uncertainty about how to interpret monoclonal protein testing and bone scintigraphy outside specialized centers.

Addressing these gaps requires a tiered diagnostic approach aligned with resource availability. At the primary care or community hospital level, clinical red flags, ECG, echocardiography with GLS, and complete monoclonal protein screening can identify patients who merit referral. At the tertiary cardiology center, bone scintigraphy with SPECT/SPECT-CT and complete monoclonal protein screening enables non-biopsy diagnosis in appropriately selected monoclonal-negative patients. At specialized amyloidosis centers, CMR with T1 mapping and ECV, genetic testing, and biopsy with mass spectrometry provide definitive characterization when non-invasive results are discordant or monoclonal testing is abnormal.

The diagnostic pathway is straightforward in most cases: suspect the diagnosis based on clinical history (CTS, spinal stenosis, low-voltage hypertrophy), screen with simultaneous bone scintigraphy and monoclonal protein testing, and initiate treatment upon confirmation. The overlap with AL amyloidosis mandates careful attention to monoclonal screening results, but the non-biopsy diagnostic algorithm is highly effective for typical ATTR-CM.

The therapeutic landscape is expanding rapidly. Tafamidis remains first-line therapy. Acoramidis and vutrisiran now offer additional treatment options. The best outcomes are achieved with early treatment, which depends on early diagnosis.

Several promising developments are on the horizon. First, next-generation gene silencing approaches (CRISPR-Cas9 *in vivo* editing, NTLA-2001) have demonstrated one-dose durability in ATTRv polyneuropathy and are being investigated for cardiac indications. Second, artificial intelligence-based algorithms for automated echocardiographic detection of apical sparing and ECG-based machine learning screening tools are under active development and could enable population-level screening. Third, ongoing trials are evaluating combination therapies (stabilizer plus gene silencer) for advanced-stage disease, as well as oral small-molecule stabilizers with improved bioavailability. Long-term safety data on sequential and combination TTR-targeted therapy will be critical as treatment options continue to expand.

For general cardiology practice, widely available tests can identify patients who warrant referral: electrocardiography for low voltage with left ventricular hypertrophy, echocardiography with GLS for relative apical sparing, and complete monoclonal protein screening. These tests do not replace confirmatory imaging or tissue typing, but they can move appropriate patients into a definitive diagnostic pathway.

By integrating targeted case-finding into the evaluation of selected high-risk HFpEF and unexplained hypertrophic phenotypes, general cardiologists can shorten the diagnostic gap in ATTR-CM while avoiding overdiagnosis and inappropriate population-level screening. The diagnostic tools are increasingly available; the rate-limiting step remains clinical awareness and correct interpretation of monoclonal testing and nuclear imaging.

## References

[1] Falk RH. Diagnosis and management of the cardiac amyloidoses. Circulation. (2005) 112(13):2047–60. 10.1161/CIRCULATIONAHA.104.489187 16186440

[2] Wechalekar AD Gillmore JD Hawkins PN. Systemic amyloidosis. Lancet. (2016) 387(10038):2641–54. 10.1016/S0140-6736(15)01274-X 26719234

[3] Ruberg FL Grogan M Hanna M Kelly JW Maurer MS. Transthyretin amyloid cardiomyopathy: JACC state-of-the-art review. J Am Coll Cardiol. (2019) 73(21):2722–7. 31171094 10.1016/j.jacc.2019.04.003 PMC6724183

[4] Connors LH Sam F Skinner M Salinaro F Sun F Ruberg FL. Heart failure resulting from age-related cardiac amyloid disease associated with wild-type transthyretin: a prospective, observational cohort study. Circulation. (2016) 133(3):282–90. 10.1161/CIRCULATIONAHA.115.018852 26660282 PMC4718760

[5] Maurer MS Hanna M Grogan M Dispenzieri A Witteles R Drachman B. Genotype and phenotype of transthyretin cardiac amyloidosis: tHAOS registry. J Am Coll Cardiol. (2016) 68(2):161–72. 27386769 10.1016/j.jacc.2016.03.596 PMC4940135

[6] Grogan M Scott CG Kyle RA Zeldenrust SR Gertz MA Lin G. Natural history of wild-type transthyretin cardiac amyloidosis and risk stratification using a novel staging system. J Am Coll Cardiol. (2016) 68(10):1014–20. 10.1016/j.jacc.2016.06.033 27585505

[7] González-López E Gallego-Delgado M Guzzo-Merello G De Haro-Del Moral FJ Cobo-Marcos M Robles C. Wild-type transthyretin amyloidosis as a cause of heart failure with preserved ejection fraction. Eur Heart J. (2015) 36(38):2585–94. 10.1093/eurheartj/ehv338 26224076

[8] Castaño A Narotsky DL Hamid N Khalique OK Morgenstern R DeLuca A. Unveiling transthyretin cardiac amyloidosis and its predictors among elderly patients with severe aortic stenosis undergoing transcatheter aortic valve replacement. Eur Heart J. (2017) 38(38):2879–87. 10.1093/eurheartj/ehx350 PMC5837725 29019612

[9] Scully PR Patel KP Treibel TA Thornton GD Hughes RK Chadalavada S. Prevalence and outcome of dual aortic stenosis and cardiac amyloid pathology in patients referred for transcatheter aortic valve implantation. Eur Heart J. (2020) 41(29):2759–67. 10.1093/eurheartj/ehaa170 32267922 PMC7395329

[10] González-López E Gallego-Delgado M Guzzo-Merello G De Haro-Del Moral FJ Cobo-Marcos M Robles C. Wild-type transthyretin amyloidosis as a cause of heart failure with preserved ejection fraction. Eur Heart J. (2015) 36(38):2585–94. 10.1093/eurheartj/ehv338 26224076

[11] Maurer MS Elliott P Comenzo R Semigran M Rapezzi C. Addressing common questions encountered in the diagnosis and management of cardiac amyloidosis. Circulation. (2017) 135(14):1357–71. 28373528 10.1161/CIRCULATIONAHA.116.024438 PMC5392416

[12] Lane T Fontana M Martinez-Naharro A Quarta CC Whelan CJ Petrie A. Natural history, quality of life, and outcome in cardiac transthyretin amyloidosis. Circulation. (2019) 140(1):16–26. 10.1161/CIRCULATIONAHA.118.038169 31109193

[13] Griffin JM Rosenthal JL Grodin JL Maurer MS Grogan M Cheng RK. ATTR amyloidosis: current and emerging management strategies. JACC CardioOncol. (2021) 3(4):488–505. 10.1016/j.jaccao.2021.09.005 34729521 PMC8543085

[14] Moñivas Palomero V Durante-Lopez A Sanabria MT Cubero JS González-Mirelis J Lopez-Ibor JV. Role of right ventricular strain measured by two-dimensional echocardiography in the diagnosis of cardiac amyloidosis. J Am Soc Echocardiogr. (2019) 32(7):845–53. 10.1016/j.echo.2019.03.005 31078369

[15] García-Pavía P García-Pinilla JM Lozano-Bahamonde A Yun S García-Quintana A Gavira-Gómez JJ. Prevalence of transthyretin cardiac amyloidosis in patients with heart failure with preserved ejection fraction: the PRACTICA study. Rev Esp Cardiol (Engl Ed). (2025) 78(4):301–10. 10.1016/j.rec.2024.07.005 39089574

[16] Garcia-Pavia P Damy T Piriou N Barriales-Villa R Cappelli F Bahus C. Prevalence and characteristics of transthyretin amyloid cardiomyopathy in hypertrophic cardiomyopathy. ESC Heart Fail. (2024) 11(6):4314–24. 10.1002/ehf2.14971 39210606 PMC11631301

[17] Sekijima Y Uchiyama S Tojo K Shimizu Y Yoshida T Misumi Y. High prevalence of wild-type transthyretin deposition in patients with idiopathic carpal tunnel syndrome: a common cause of carpal tunnel syndrome in the elderly. J Neurol. (2011) 258(11):1789–91. 10.1016/j.humpath.2011.03.004 21733562

[18] Westin O Fosbøl EL Butt JH. Transthyretin amyloidosis and carpal tunnel syndrome: a population-based study. J Am Coll Cardiol. (2022) 79(18):1781–92. 10.1016/j.jacc.2022.02.04535483761

[19] Mundayat R Stewart M Alvir J. The effectiveness of tafamidis in TTR-FAP: analysis of the THAOS registry. Neurol Ther. (2022) 11(1):373–90. 10.1007/s40120-021-00318-235064907

[20] Maurer MS Schwartz JH Gundapaneni B Elliott PM Merlini G Waddington-Cruz M. Tafamidis treatment for patients with transthyretin amyloid cardiomyopathy (ATTR-ACT). N Engl J Med. (2018) 379(11):1007–16. 10.1056/NEJMoa1805689 30145929

[21] Elliott P Drachman BM Gottlieb SS. Long-term survival with tafamidis in patients with transthyretin amyloid cardiomyopathy. Circ Heart Fail. (2022) 15(1):e008193. 10.1161/CIRCHEARTFAILURE.121.008193 34923848 PMC8763250

[22] Perfetto F Zampieri M Bandini G Fedi R Tarquini R Santi R. Transthyretin cardiac amyloidosis: a cardio-orthopedic disease. Biomedicines. (2022) 10(12):3226. 10.3390/biomedicines10123226 36551982 PMC9775219

[23] Donnelly JP Hanna M. Cardiac amyloidosis: an update on diagnosis and treatment. Cleve Clin J Med. (2017) 84(12 suppl 3):12–26. 10.3949/ccjm.84.s3.02 29257735

[24] Nakagawa M Sekijima Y Yazaki M. Carpal tunnel syndrome as the initial symptom of wild-type ATTR amyloidosis. Amyloid. (2016) 23(1):58–63. 10.3109/13506129.2016.1136800 26852880

[25] Sueyoshi T Ueda M Jono H Irie H Sei A Ide J. Wild-type transthyretin-derived amyloidosis in various ligaments and tendons. Hum Pathol. (2011) 42(11):1752–8. 10.1016/j.humpath.2010.11.017 21334722

[26] Westermark P Westermark GT Suhr OB Berg S. Transthyretin-derived amyloid in lumbar spinal stenosis. Ups J Med Sci. (2014) 119(3):223–8. 10.3109/03009734.2014.920450 24620715 PMC4116761

[27] Geller HI Singh A Alexander KM Mirto TM Falk RH. Association between ruptured distal biceps tendon and wild-type transthyretin cardiac amyloidosis. JAMA. (2017) 318(10):962–3. 10.1001/jama.2017.9236 28898370 PMC5818850

[28] Rao V Diez J Gustafsson F Mentz R Senni M Jankowska E. Biceps tendon rupture: a clue for cardiac amyloidosis. J Card Fail. (2023) 29(3):397–402. 10.1016/j.cardfail.2022.10.431 36529314

[29] Cappelli F Zampieri M Fumagalli C Nardi G Del Monaco G Matucci Cerinic M. Tenosynovial complications identify TTR cardiac amyloidosis among patients with hypertrophic cardiomyopathy phenotype. J Intern Med. (2021) 289(6):831–9. 10.1111/joim.13200 33615623

[30] Nitsche C Scully PR Patel KP. Prevalence and outcomes of concomitant aortic stenosis and cardiac amyloidosis. J Am Coll Cardiol. (2021) 77(2):128–39. 10.1016/j.jacc.2020.11.002 33181246 PMC7805267

[31] Galat A Guellich A Bodez D Slama M Dijos M Zeitoun DM. Aortic stenosis and transthyretin cardiac amyloidosis: the chicken or the egg? Eur Heart J. (2016) 37(47):3525–32. 10.1093/eurheartj/ehw033 26908951

[32] Adams D Koike H Slama M Coelho T. Hereditary transthyretin amyloidosis: a model of medical progress for a fatal disease. Nat Rev Neurol. (2019) 15(7):387–404. 10.1038/s41582-019-0195-5 31209302

[33] Gonzalez-Duarte A Berk JL Quan D. Autonomic dysfunction in transthyretin amyloidosis. Auton Neurosci. (2022) 237:102905. 10.1016/j.autneu.2021.102905 34800845 PMC9280330

[34] Phelan D Collier P Thavendiranathan P Popović ZB Hanna M Plana JC. Relative apical sparing of longitudinal strain using two-dimensional speckle-tracking echocardiography is both sensitive and specific for the diagnosis of cardiac amyloidosis. Heart. (2012) 98(19):1442–8. 10.1136/heartjnl-2012-302353 22865865

[35] Senapati A Sperry BW Grodin JL Kusunose K Thavendiranathan P Jaber W. Prognostic implication of relative regional strain ratio in cardiac amyloidosis. Heart. (2016) 102(10):778–84. 10.1136/heartjnl-2015-308657 26830665

[36] Robin G Cognet T Bouisset F Cariou E Méjean S Pradel S. Value of longitudinal strain to identify wild-type transthyretin amyloidosis in patients with aortic stenosis. Circ J. (2021) 85(8):1293–301. 10.1253/circj.CJ-20-1064 33980765

[37] Murtagh B Hammill SC Gertz MA Kyle RA Tajik AJ Grogan M. Electrocardiographic findings in primary systemic amyloidosis and biopsy-proven cardiac involvement. Am J Cardiol. (2005) 96(12):1751–9. 10.1016/j.amjcard.2005.07.020 15695149

[38] Perugini E Guidalotti PL Salvi F Cooke TE Rizzati F Lunghetti S. Noninvasive etiologic diagnosis of cardiac amyloidosis using 99mTc-3,3-diphosphono-1,2-propanodicarboxylic acid scintigraphy. J Am Coll Cardiol. (2005) 46(6):1076–84. 10.1016/j.jacc.2005.05.073 16168294

[39] Gillmore JD Maurer MS Falk RH Merlini G Damy T Dispenzieri A. Nonbiopsy diagnosis of cardiac transthyretin amyloidosis. Circulation. (2016) 133(24):2404–12. 10.1161/CIRCULATIONAHA.116.021612 27143678

[40] Dorbala S Ando Y Bokhari S Dispenzieri A Falk RH Ferrari VA. ASNC/AHA/ASE/EANM/HFSA/ISA/SCMR/SNMMI expert consensus recommendations for multimodality imaging in cardiac amyloidosis. J Nucl Cardiol. (2019) 26(6):2065–123. 10.1007/s12350-019-01760-6 31468376

[41] Dorbala S Cuddy S Falk RH. How to image cardiac amyloidosis: a practical approach. JACC Cardiovasc Imaging. (2020) 13(6):1368–83. 10.1016/j.jcmg.2019.07.015 31607664 PMC7148180

[42] Muchtar E Gertz MA Kumar SK Lacy MQ Dingli D Buadi FK. Improved outcomes for newly diagnosed AL amyloidosis between 2000 and 2014: cracking the glass ceiling of early death. Blood. (2017) 129(15):2111–9. 10.1182/blood-2016-11-751628 28126928 PMC5391625

[43] Vrana JA Gamez JD Madden BJ Theis JD Bergen 3 Dogan A. Classification of amyloidosis by laser microdissection and mass spectrometry-based proteomic analysis in clinical biopsy specimens. Blood. (2009) 114(24):4957–9. 10.1182/blood-2009-07-230722 19797517

[44] Martinez-Naharro A Kotecha T Norrington K Boldrini M Rezk T Quarta C. Native T1 and extracellular volume in transthyretin amyloidosis. JACC Cardiovasc Imaging. (2019) 12(5):810–9. 10.1016/j.jcmg.2018.02.006 29550324

[45] Banypersad SM Sado DM Flett AS Gibbs SDJ Pinney JH Maestrini V. Quantification of myocardial extracellular volume fraction in systemic AL amyloidosis: an equilibrium contrast CMR study. Circ Cardiovasc Imaging. (2013) 6(1):34–9. 10.1161/CIRCIMAGING.112.978627 23192846

[46] Martinez-Naharro A Treibel TA Abdel-Gadir A Bulluck H Zumbo G Knight DS. Magnetic resonance in transthyretin cardiac amyloidosis. J Am Coll Cardiol. (2017) 70(1):70–7. 10.1016/j.jacc.2017.05.053 28728692

[47] Arbelo E Protonotarios A Gimeno JR Arbustini E Barriales-Villa R Basso C. 2023 ESC guidelines for the management of cardiomyopathies. Eur Heart J. (2023) 44(37):3503–626. 10.1093/eurheartj/ehad194 37622657

[48] Maurer MS Bokhari S Damy T Dorbala S Drachman BM Fontana M. Expert consensus recommendations for the suspicion and diagnosis of transthyretin cardiac amyloidosis. Circ Heart Fail. (2019) 12(9):e006075. 10.1161/CIRCHEARTFAILURE.119.006075 31480867 PMC6736650

[49] Kastritis E Dimopoulos MA. Prognosis and risk assessment in AL amyloidosis – there and back again. Br J Haematol. (2017) 178(3):379–89. 10.1111/bjh.14587 28437006

[50] Gillmore JD Damy T Fontana M Hutchinson M Lachmann HJ Martinez-Naharro A. A new staging system for cardiac transthyretin amyloidosis. Eur Heart J. (2018) 39(30):2799–806. 10.1093/eurheartj/ehx589 29048471

[51] Rosenblum H Masri A Narotsky DL. Tafamidis treatment across the spectrum of transthyretin cardiac amyloidosis disease severity. JAMA Cardiol. (2023) 8(8):761–9. 10.1001/jamacardio.2023.1783

[52] Gillmore JD Judge DP Cappelli F. Efficacy and safety of acoramidis in transthyretin amyloid cardiomyopathy (ATTRibute-CM). N Engl J Med. (2024) 390(2):132–42. 10.1056/NEJMoa2309138 38197816

[53] Fontana M Berk JL Gillmore JD Witteles RM Grogan M Drachman B. Vutrisiran in patients with transthyretin amyloidosis with cardiomyopathy (HELIOS-B). N Engl J Med. (2025) 392(5):447–58. 10.1056/NEJMoa2409134 39213194

[54] Maurer MS Kale P Fontana M. Patisiran in patients with transthyretin cardiac amyloidosis: the APOLLO-B trial. N Engl J Med. (2023) 389(25):2337–47. 10.1056/NEJMoa2300772 PMC10757426 37888916

[55] Conceição I Berk JL Weiler M Kowacs PA Dasgupta NR Khella S. Switching from inotersen to eplontersen in patients with hereditary transthyretin-mediated amyloidosis with polyneuropathy: analysis from NEURO-TTRansform. J Neurol. (2024) 271(10):6655–66. 10.1007/s00415-024-12616-6 PMC11447117 39138650

[56] Gillmore JD Gane E Taubel J Kao J Fontana M Maitland ML. CRISPR-Cas9 *in vivo* gene editing for transthyretin amyloidosis. N Engl J Med. (2021) 385(6):493–502. 10.1056/NEJMoa2107454 34215024

[57] Fontana M García-Pavía P Grogan M Shah SJ Engelmann MDM Hovingh GK. Coramitug, a humanized monoclonal antibody for the treatment of transthyretin amyloid cardiomyopathy: a phase 2, randomized, multicenter, double-blind, placebo-controlled trial. Circulation. (2026) 153(4):214–25. 10.1161/CIRCULATIONAHA.125.077304 41212997 PMC12851541

[58] Kahr PC Aus Dem Siepen F Lairez O Donal E Damy T Kristen AV. Cliramitug for depletion of cardiac amyloid transthyretin: long-term follow-up of the NI006-101 trial. Nat Med. (2026) 32(7):2639–46. 10.1038/s41591-026-04487-3 42362866 PMC13375531

[59] Ericzon BG Wilczek HE Larsson M. Liver transplantation for hereditary transthyretin amyloidosis: 20-year results. Transplantation. (2015) 99(9):1847–54. 10.1097/TP.0000000000000698 26308415

[60] Berk JL Suhr OB Obici L Sekijima Y Zeldenrust SR Yamashita T. Repurposing diflunisal for familial amyloid polyneuropathy: a randomized clinical trial. JAMA. (2013) 310(24):2658–67. 10.1001/jama.2013.283815 24368466 PMC4139164

[61] Damy T Costes B Hagège AA Donal E Eicher JC Slama M. Prevalence and clinical phenotype of hereditary transthyretin cardiac amyloidosis in patients with increased left ventricular wall thickness. Eur Heart J. (2016) 37(23):1826–34. 10.1093/eurheartj/ehv583 26537620

[62] Pagourelias ED Mirea O Duchenne J Van Cleemput J Delforge M Bogaert J. Echo parameters for differential diagnosis in cardiac amyloidosis: a head-to-head comparison of deformation and nondeformation parameters. Circ Cardiovasc Imaging. (2017) 10(3):e005588. 10.1161/CIRCIMAGING.116.005588 28298286

